# Diproline-induced resistance to parasitic nematodes in the same and subsequent rice generations: Roles of iron, nitric oxide and ethylene

**DOI:** 10.3389/fpls.2023.1112007

**Published:** 2023-02-07

**Authors:** Jonas De Kesel, Eli Bonneure, Michael Frei, Tim De Meyer, Sven Mangelinckx, Tina Kyndt

**Affiliations:** ^1^ Department of Biotechnology, Faculty of Bioscience Engineering, Ghent University, Ghent, Belgium; ^2^ Department of Green Chemistry and Technology, Faculty of Bioscience Engineering, Ghent University, Ghent, Belgium; ^3^ Department of Agronomy and Crop Physiology, Institute for Agronomy and Plant Breeding, Justus Liebig University Giessen, Giessen, Germany; ^4^ Department of Data Analysis and Mathematical Modelling, Faculty of Bioscience Engineering, Ghent University, Ghent, Belgium

**Keywords:** induced resistance (IR), defense priming, rice (*Oryza sativa*), rice root-knot nematode (*Meloidogyne graminicola*), diproline (cyclo(L-Pro-L-Pro)), Fe homeostasis, transgenerational IR

## Abstract

Induced resistance (IR) is a plant phenotype characterized by lower susceptibility to biotic challenges upon elicitation by so-called IR stimuli. Earlier, we identified diproline (*cyclo*(l-Pro-l-Pro)) as IR stimulus that protects rice (*Oryza sativa*) against the root-knot nematode *Meloidogyne graminicola* (*Mg*). In the current study, detailed transcriptome analyses at different time points, and under uninfected and nematode-infected conditions revealed that this rice IR phenotype is correlated with induction of genes related to iron (Fe), ethylene (ET) and reactive oxygen species (ROS)/reactive nitrogen species (RNS) metabolism. An infection experiment under Fe limiting conditions confirmed that diproline-IR is only effective under optimal Fe supply. Although total root Fe levels were not affected in diproline-treated plants, phytosiderophore secretion was found to be induced by this treatment. Experiments on mutant and transgenic rice lines impaired in ET or ROS/RNS metabolism confirmed that these metabolites are involved in diproline-IR. Finally, we provide evidence for transgenerational inheritance of diproline-IR (diproline-TIR), as two successive generations of diproline-treated ancestors exhibited an IR phenotype while themselves never being exposed to diproline. Transcriptome analyses on the offspring plants revealed extensive overlap between the pathways underpinning diproline-IR and diproline-TIR. Although diproline induces significant systemic changes in global DNA methylation levels early after treatment, such changes in DNA methylation were not detected in the descendants of these plants. To our knowledge, this is the first report of TIR in rice and the first transcriptional assessment of TIR in monocots.

## Introduction

Rice is one of the most important crops grown worldwide. Recent agronomic practices in rice cultivation include a shift from flooded ‘paddy fields’ to aerobic growth conditions with the aim to reduce greenhouse gas emission and water consumption. This comes with major challenges in disease control (Kreye et al., 2009). More specifically, root pathogens – such as the root-knot nematode *Meloidogyne graminicola* (*Mg*) – that were previously controlled by flooding now gain in terms of incidence and disease severity, thereby severely impacting rice yield (De Waele and Elsen, 2007; Kreye et al., 2009; Priyanka et al., 2012; Sharma et al., 2016). Ecological concerns have concomitantly led to progressive banning of chemical nematicides (Mantelin et al., 2017; Oka, 2020). Hence the demand for alternative control strategies has strongly risen (Dias-Arieira et al., 2013; Mantelin et al., 2017). One of the potential alternatives is induced resistance (IR), a plant phenotype evoked by IR stimuli that is characterized by enhanced immunity to biotic challenges in the same generation, and in some cases even reported to provide protection to subsequent generations (Mauch-Mani et al., 2017; De Kesel et al., 2021). IR is typified by induction of defense genes and/or defense priming, the latter being a stronger defense response upon pathogen attack (De Kesel et al., 2021). Transgenerational IR (TIR) has been demonstrated in β-amino butyric acid (BABA)-treated arabidopsis (*Arabidopsis thaliana*; Slaughter et al., 2012), as well as in saccharin- or benzothiadiazole-treated barley plants (*Hordeum vulgare*) (Walters and Paterson, 2012). Specific for nematodes, de Medeiros et al. (2017) demonstrated TIR to *Meloidogyne javanica* in the progeny of *Trichoderma atroviride*-inoculated tomato (*Solanum lycopersicum*) plants. This phenotype was linked to transgenerational stimulation of auxin-induced reactive oxygen species (ROS) production. How IR is transmitted across generations is far from completely unraveled although there are various indications that epigenetic mechanisms, such as DNA methylation, are involved (Luna and Ton, 2012; López Sánchez et al., 2016; Stassen et al., 2018; Kuźnicki et al., 2019; López Sánchez et al., 2021). For example, BABA induces transient DNA hypermethylation in treated potato leaves, which later reverts into hypomethylation, and this hypomethylation pattern slightly persists in the subsequent generation (Kuźnicki et al., 2019).

The diketopiperazine diproline (*cyclo*(l-Pro-l-Pro)) is known to act as attraction pheromone for the diatom *Seminavis robusta* and has been detected in various other species, often as constituent of pheromonal secretions (Bonneure et al., 2021). Recently, we demonstrated that foliar diproline treatment leads to systemic IR against *Mg* in rice grown under aerobic conditions (De Kesel et al., 2020). This study extended previous observations of Wu et al. (2017b) who demonstrated that diproline-treated tobacco (*Nicotiana benthamiana*) is less susceptible to the leaf pathogens *Phytophthora nicotianae* and tobacco mosaic virus. In tobacco, diproline treatment leads to accumulation of salicylic acid (SA) and stomatal closure, which is associated with increased cytosolic calcium and nitric oxide (NO) in guard cells (Wu et al., 2017b). To date, the underlying mechanism of diproline-IR in monocots and/or against belowground pathogens has not yet been studied. In case of other IR stimuli that protects rice against *Mg*, such as ascorbate oxidase (AO) (Singh et al., 2020), and thiamine (Huang et al., 2016) the reduced susceptibility of the plants is based on early accumulation of hydrogen peroxide (H_2_O_2_), as well as activation of hormonal pathways such as ethylene (ET) and jasmonic acid (JA). In general, the JA pathway – activated by ET – has a central role in systemic rice defense against *Mg* (Nahar et al., 2011).

Iron (Fe) is known to play an important role in plant-pathogen interactions (Lemanceau et al., 2009; Expert et al., 2012; Naranjo-Arcos and Bauer, 2016). It is required in sufficient quantities to activate plant immune responses, as well as pathogen virulence (Aznar and Dellagi, 2015). Various compounds secreted by plant roots, such as coumarins, contribute to Fe acquisition while also having an anti-pathogenic function (Stringlis et al., 2019). Fe is essential in the generation of specific ROS *via* the Fenton reaction (Mittler, 2017), especially for grasses (Poaceae) where recruitment of Fe to infection sites to exploit its redox chemistry is known to be a critical immune response (Herlihy et al., 2020). A form of programmed cell death (PCD) named ferroptosis has been shown to be the causative mechanism for hypersensitive response-associated cellular suicide in rice upon interaction with *Magnaporthe oryzae* (Dangol et al., 2019). This form of PCD is marked by accumulation of Fe ions that convert H_2_O_2_ in highly cytotoxic hydroxyl radicals *via* the Fenton reaction (Mittler, 2017; Herlihy et al., 2020). The role of Fe in IR establishment has mostly been studied in dicots, in particular in arabidopsis upon colonization with plant growth-promoting rhizobacteria and fungi. Transcription factor *AtMYB72* – involved in Fe deficiency responses – is induced in arabidopsis roots upon *Pseudomonas simiae* WCS417r-IR establishment (Zamioudis et al., 2014; Pieterse et al., 2021). This triggers root secretion of Fe mobilizing scopoletin molecules, leading to enrichment of beneficial microorganisms, growth stimulation and immunity benefits for the plant (Stringlis et al., 2018; Pieterse et al., 2021). Moreover, Koen et al. (2014) reported that root drenching of arabidopsis with BABA leads to disturbed expression of Fe homeostasis-related genes in the shoots. As no differences in Fe assimilation were detected, the authors concluded that through Fe chelation, BABA may affect *in planta* Fe availability or distribution (Koen et al., 2014). Finally, Fe homeostasis disturbances *per se* have recently been shown to be potent triggers for the establishment of IR in arabidopsis against various pathogens (Trapet et al., 2021).

In dicots, Fe homeostasis, plant innate immunity and IR establishment seem to converge on some common signaling molecules (Aznar et al., 2015; Verbon et al., 2017; Romera et al., 2019; Herlihy et al., 2020; Trapet et al., 2021). García et al. (2011), describes how Fe deficiency in dicot roots drives the production of ET and NO, both known to be essential mediators of plant immunity and thought to be the main inducers of Fe acquisition gene transcription (García et al., 2011). Accordingly, the models of Romera et al. (2019) and Herlihy et al. (2020) indicate that NO and ET, triggered upon Fe deficiency responses and/or defense stimulation, induce phytohormone biosynthesis, as well as ROS production and detoxification *via* the glutathione-ascorbate redox system. However, the link between plant immunity and Fe, NO and ET is poorly studied in monocot plants and even shows divergence when comparing both clades. JA, for instance, is often described as a negative regulator of Fe import for dicots (Aznar et al., 2015; Verbon et al., 2017; Trapet et al., 2021), whereas it facilitates early Fe deficiency responses in rice (Kobayashi et al., 2014). These contrasting observations have been attributed to the divergent Fe import mechanisms employed by both plant types (Garnica et al., 2018). While dicots rely on ‘strategy I Fe import’, in which Fe^3+^ is reduced to Fe^2+^ in the rhizosphere before import, graminaceous monocots use ‘strategy II Fe import’, in which Fe^3+^ is imported upon chelation in the rhizosphere with phytosiderophores (PSs) (Aznar et al., 2015; Naranjo-Arcos and Bauer, 2016; Verbon et al., 2017; Herlihy et al., 2020; Liu et al., 2021). However, rice is one of the few plants known to possess active elements of both Fe import strategies (Aznar et al., 2015; Naranjo-Arcos and Bauer, 2016; Verbon et al., 2017; Liu et al., 2021). In rice, ET has been shown to positively affect Fe import (Wu et al., 2011). Although it is known that ET (Nahar et al., 2011), JA (Nahar et al., 2011; Singh et al., 2020) and redox reactions through the glutathione-ascorbate system (Singh et al., 2020) fulfil crucial roles in protection of rice against infection by *Mg*, the role of Fe and NO in rice defense against nematodes is largely unknown.

In this study, we investigated the mode-of-action of diproline-IR in rice against *Mg*. Based on transcriptome analyses, we detected a significant role for Fe homeostasis, linked to ET- and NO-mediated signaling, in diproline-IR against *Mg* in rice. The activation of these pathways was independently validated by biochemical measurements. Moreover, the IR phenotype induced by diproline was shown to be inherited by two successive untreated generations, as these descendants remained significantly less susceptible to *Mg*. This provides conclusive evidence for diproline-TIR. RNA seq on the descendants revealed that the physiological pathways affected in TIR plants were strongly overlapping with those induced in diproline-treated ancestral plants. Finally, DNA methylation analyses showed that diproline quickly induces global DNA hypomethylation, but that this genome-wide hypomethylation profile is not transmitted to the descendants.

## Results

### Foliar diproline treatment leads to changes in Fe homeostasis, nitrogen metabolism and redox chemistry in rice roots

In a previous paper, we reported on the reduced susceptibility of rice plants to *Meloidogyne graminicola* (*Mg)* after foliar treatment with 500 µM diproline (De Kesel et al., 2020). Although this observation can be explained by activation of systemic IR, an alternative hypothesis was first investigated here. Potentially, diproline could be transported inside the plant towards the roots and/or run-off could end up in the substrate, where it could have a nematicidal effect. To evaluate this possibility, diproline presence inside plant roots was investigated at four days post foliar treatment (4 dpt). No diproline could be detected in rice roots (**Supporting Information 2**). In addition, exposure of nematodes to 500 µM diproline resulted in only minor nematicidal effects (**Supporting Information 3**). These results suggest that diproline particularly reduces rice susceptibility to *Mg via* systemic IR.

To study the systemic responses induced by foliar diproline treatment, transcriptional changes were investigated in rice roots at 1 dpt and 4 dpt *via* RNA seq. Indeed, defense responses can be triggered quickly upon contact with an IR stimulus, even before presence of a challenging pathogen (De Kesel et al., 2021). In addition, IR can lead to primed defense responses, which are only apparent after infection (De Kesel et al., 2021). For this reason, RNA seq was also performed on root systems infected with the root-knot nematode *Mg*. Mock-treated control plants were evaluated at three days post *Mg* inoculation (3 dpi) and compared to plants that were diproline-pretreated and *Mg*-inoculated (4/3 dpt/i).

At 1 dpt, no statistically significant differentially expressed genes (DEGs) were identified upon diproline treatment. However, at 4 dpt, 448 DEGs were found between mock-treated and diproline-treated plants. Gene ontology (GO)-analyses showed enrichment for genes related to nitrogen and/or nitric oxide (NO) metabolism among the 122 significantly upregulated DEGs at 4 dpt (**Table 1**), among which nitrate reductase (*OsNIA1*). Among the 326 downregulated DEGs at 4 dpt, GO-terms related to carbohydrate metabolism, cell wall related processes, trehalose biosynthesis and photosynthesis were enriched (**Supporting information 4**). Mapman analyses revealed that amino acid and cell wall metabolism, jasmonate (JA) metabolism and the glutathione-ascorbate redox system were affected in diproline treated plants at this later time point (**Supporting information 4**). Although no significant DEGs were detected at 1 dpt, Mapman analyses on all expression changes (regardless of significance) revealed that redox chemistry *via* the glutathione-ascorbate cycle was already slightly affected at this early time point (**Table 1**). When comparing 4/3 dpt/i to 3 dpi plants to evaluate if some defense pathways were primed, the Mapman bins ‘peroxidases’ and ‘secondary metabolism *via* the phenylpropanoid pathway’ were found to respond stronger upon nematode attack than in mock-treated plants (**Table 1**). All transcript levels, as well as complete GO-enrichment and Mapman analyses are shown in **Supporting Information 4**.

**Table 1:** Diproline-induced resistance in rice is associated with transcriptional induction of various defense-related compounds and pathways.

1 dpt *vs*. mock-treated plants		
GO term/Mapman bin	Term/bin ID	p-value
Redox reactions *via* the glutathione-ascorbate cycle	Bin 21.2	4.77E-3

4 dpt *vs*. mock-treated plants		
GO term/Mapman bin	Term/bin ID	p-value
Nitrate metabolic process	GO:0042126	2.25E-2
Nitrate assimilation	GO:0042128	2.25E-2
Reactive nitrogen species metabolic process	GO:2001057	2.25E-2
Nitrogen cycle metabolic process	GO:0071941	4.98E-2
Redox reactions *via* the glutathione-ascorbate cycle	Bin 21.2	4.58E-2
Jasmonate metabolism	Bin 17.7	4.99E-2

Primed response: 4/3 dpt/i *vs*. 3 dpi		
GO term/Mapman bin	Term/bin ID	p-value
Peroxidases	Bin 26.12	2.65E-8
Secondary metabolism - phenylpropanoids	Bin 16.2	4.85E-4

Gene expression levels were assessed in rice roots *via* RNA seq one and four days post foliar treatment with 500 µM diproline (1 dpt and 4 dpt, respectively), three days post nematode inoculation (3 dpi) and upon a combination of diproline treatment and nematode inoculation (4/3 dpt/i). Listed is a selection of plant immunity-associated gene ontology (GO) terms that were significantly enriched among the genes significantly induced in the data sets. MapMan bins that were found to be enriched using a WRS test on all expression levels are also shown. Complete lists of GO-enrichment and Mapman analyses can be found in **Supporting Information 4**.

**Figure 1:**
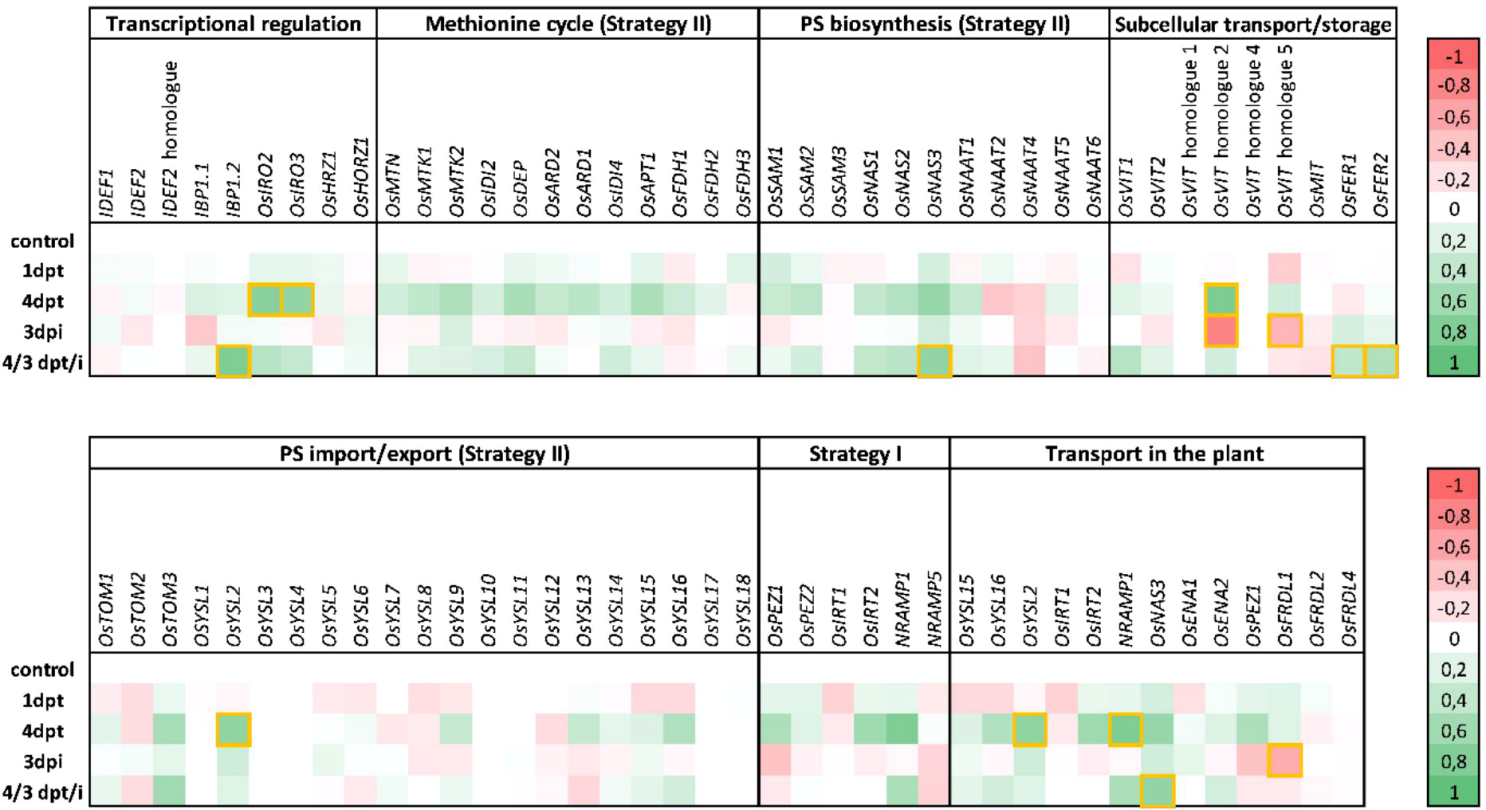
Diproline-induced resistance in rice is associated with general upregulation of iron homeostasis genes. Fe-homeostasis genes were selected based on a thorough literature review (Lemanceau et al., 2009; Expert et al., 2012; Kobayashi et al., 2014; Aznar and Dellagi, 2015; Aznar et al., 2015; Naranjo-Arcos and Bauer, 2016; Verbon et al., 2017). The figure shows RNA seq based relative expression levels of these genes in rice roots at one and four days post foliar treatment with 500 µM diproline (1 dpt and 4 dpt, respectively), three days post *Meloidogyne graminicola* inoculation (3 dpi) and upon a combination of diproline treatment and nematode inoculation (4/3 dpt/i). Relative expression levels are expressed as log2fold changes compared to the same-aged, mock-treated uninoculated plants control plants. Green and red represent transcriptional induction and repression, respectively. Yellow-boxed cells indicate statistically significant up- or downregulation (p<0.05). PS = phytosiderophore. Detailed per gene transcription levels can be found in **Supporting Information 4**.

Manual evaluation of the genes significantly induced four days after diproline treatment, revealed that five of them were related to Fe homeostasis and transport: *VIT1*, *IRO2*, *IRO3*, *NRAMP1* and *YSL2*. As Fe homeostasis-related genes are often poorly annotated, we performed an additional literature review to adequately compile the rice genes that are involved in Fe metabolism (Lemanceau et al., 2009; Expert et al., 2012; Kobayashi et al., 2014; Aznar and Dellagi, 2015; Aznar et al., 2015; Naranjo-Arcos and Bauer, 2016; Verbon et al., 2017). Differential expression levels of these manually annotated genes in our RNA seq data sets is represented in **Figure 1**. This additional analysis revealed multiple genes related to Fe transport or homeostasis to be induced by diproline treatment, mainly at 4 dpt. Although not always statistically significant, upstream processes such as transcriptional regulation and biosynthesis of methionine – the precursor for strategy II phytosiderophores (PSs) – were generally induced, as well as genes related to type I and type II Fe import, and downstream processes such as Fe transport and storage (**Figure 1**).

### Diproline-IR is dependent on Fe supply, and linked to the NO and ET pathway

**Figure 2:**
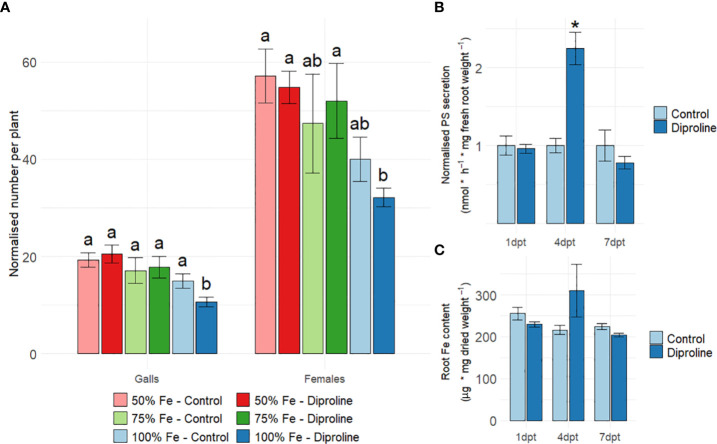
Diproline-induced resistance in rice is dependent on Fe supply. **(A)** Normalized number of galls and nematodes per plant, as assessed fourteen days after inoculation with 250 *Meloidogyne graminicola* second-stage juvenile nematodes. One day before inoculation, fourteen-days-old rice shoots were treated with water (control) or 500 µM diproline. During the entire experiment, plants were supplied with different levels of Fe, expressed relative to the optimal concentration of 75 µM (i.e. 100%) (n = 16). **(B)** Normalized phytosiderophore (PS) levels in root exudates after foliar treatment with water (control) or 500 µM diproline (n = 4). **(C)** Intracellular root Fe levels after foliar treatment with water (control) or 500 µM diproline (n = 3). **(B, C)** Plants were grown under optimal Fe supply and were uninoculated for the PS and Fe quantifications. Error bars represent the standard error of the mean. **(A–C)** Asterisks indicate significant differences upon comparison of diproline-treated plants with same-aged, mock-treated control plants. In subpanel a, treatments with different letters are significantly different (p < 0.05), according to the ANOVA and a *post-hoc* Duncan’s new multiple range test. In subpanels, b and c, asterisks indicate a significant difference from mock-treated plants according to a two-sided heteroscedastic t-test (p<0.05).

RNA seq indicated a role for Fe in diproline-IR (**Figure 1**). To further explore this, an *Mg* infection experiment was executed using rice plants that were given 50%, 75% and 100% of the optimal Fe supply for rice growth (**Supporting Information 1**). Since excessive Fe provision has dramatic effects on rice development and growth (Aung and Masuda, 2020), increased Fe supplies were not investigated. As illustrated in **Figure 2A**, diproline-IR was found to be effective only under optimal Fe availability. Indeed, only when 100% Fe was available, the number of galls was significantly reduced in diproline-treated plants in comparison to control plants, confirming earlier results that were executed under 100% Fe supply (De Kesel et al., 2020). To allow proper investigation of diproline-IR, all further experiments were executed under 100% Fe conditions. To monitor whether diproline treatment affects rice Fe uptake, PS levels were quantified in root exudates (**Figure 2B**), and endogenous root Fe levels were determined (**Figure 2C**). Whereas observed differences for root Fe levels were non-significant, PS secretion was significantly higher for diproline-treated plants at 4 dpt (p = 5.04E-3).

**Figure 3:**
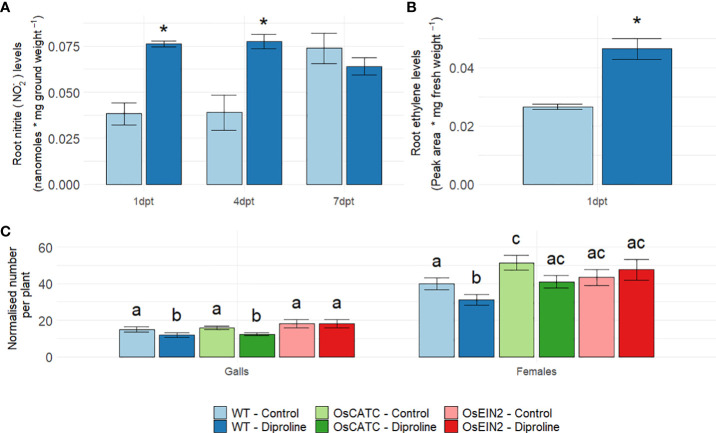
Ethylene and nitric oxide mediate diproline-induced resistance in rice. **(A)** Root nitrite $\left(NO_{2}^{-}\right)$ levels, one, four and seven days post foliar treatment with water (control) or 500 µM diproline (1 dpt, 4 dpt and 7 dpt, respectively) (n = 5). **(B)** Root ethylene levels at 1 dpt (n = 5). **(C)** Normalized number of galls and female nematodes per plant, as assessed fourteen days after inoculation with 250 *Meloidogyne graminicola* second-stage juvenile nematodes (n = 16). One day before inoculation, plants were treated with water (control) or 500 µM diproline. OsCATC is defective in CATALASE C (Os03g0131200). OsEIN2 is an RNAi-line of ETHYLENE-INSENSITIVE PROTEIN 2 (Os07g0155600). Error bars represent the standard error of the mean. **(A–C)**. In subpanels, a and b, asterisks indicate a significant difference from mock-treated plants according to a two-sided heteroscedastic t-test (p<0.05). In subpanel c, treatments with different letters are significantly different (p < 0.05), according to a non-parametric Mann-Whitney test (p<0.05).

In dicots, RNS such as NO seem to play an important and interconnecting signaling function in biotic defense responses, IR establishment and Fe homeostasis (Romera et al., 2019; Herlihy et al., 2020; Liu et al., 2021). Also in diproline-treated plants, NO-related processes (**Table 1**) were found to be induced. Therefore, the role of NO was investigated in further detail. At 1 dpt and 4 dpt, root nitrite $\left(NO_{2}^{-}\right)$ levels [*i.e.* the direct precursor of NO in the $NO_{3}^{-}-NO_{2}^{-}-NO$ biosynthesis pathway (Gupta et al., 2011)] were significantly higher in diproline-treated rice plants (p = 2.11E-3 and p = 1.24E-2, respectively) (**Figure 3A**).

A third pathway strongly induced according to our RNA seq data was the glutathione-ascorbate redox cycle (**Table 1**), which was previously shown to induce ethylene (ET) and subsequent jasmonic acid (JA) accumulation in rice roots (Singh et al., 2020). Since an interconnection between Fe, ET and immunity has been described in dicots (Romera et al., 2019; Herlihy et al., 2020), we decided to evaluate if diproline-treated plants would produce more ET. This analysis was only done at 1 dpt, since release of the gaseous ET is typically occurring quickly after treatment. Confirming our hypothesis, ET accumulation was observed in rice roots at 1 dpt (p = 9.53E-3) (**Figure 3B**), which could explain the later induction of JA-related genes (**Table 1**). To further investigate a potential causal role for NO and ET in the studied IR phenotype, an *Mg* infection experiment was conducted using rice lines defective in *CATALASE C* (*OsCATC*) (Lin et al., 2012) or impaired in ET-signaling through silencing of *ETHYLENE-INSENSITIVE PROTEIN 2* (*EIN2*) (Bailey et al., 2009), in comparison with wild-type (WT) Nipponbare plants. In contrast to Kitaake, used in previous experiments, the Nipponbare cultivar seemed to respond differently to diproline treatment: for both the numbers of females and galls, the reduction in treated plants was found to be significant. Remarkably, untreated *OsCATC* mutants accommodated significantly more females than WT plants, while diproline-IR was found to be slightly less effective in reducing female numbers in the *OsCATC* plants when compared to WT plants (**Figure 3C**). Based on the previous observations that the *OsCATC* mutant has higher endogenous H_2_O_2_ and NO levels (Lin et al., 2012; Wang et al., 2013), these data seem to indicate that ROS/RNS detoxification is important for diproline-IR. The *EIN2* RNAi line, on the other hand, was insensitive for IR establishment mediated by diproline, revealing that diproline-IR is ET-signaling dependent.

### Diproline-IR is transgenerationally inherited

**Figure 4:**
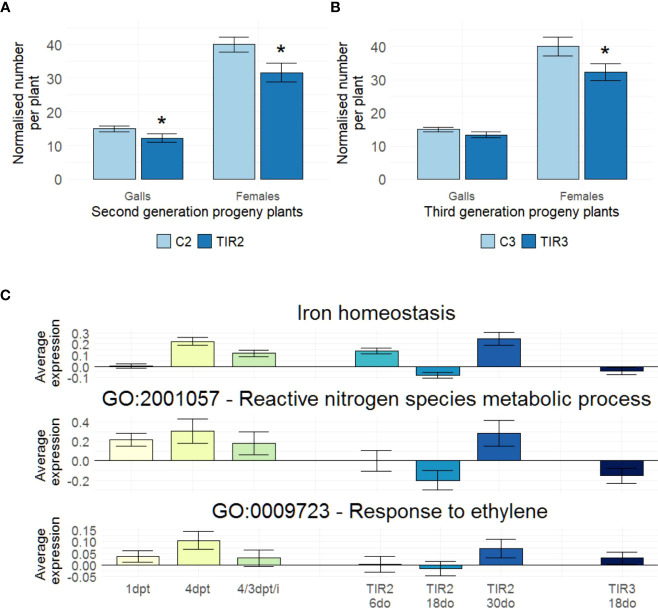
Diproline-induced resistance is inherited transgenerationally, with similar pathways affected in ancestor and progeny plants. **(A, B)** Normalized number of galls and number of females, as assessed fourteen days post inoculation with 250 *Meloidogyne graminicola* (*Mg*) second-stage juvenile nematodes for untreated **(A)** second and **(B)** third generation progeny plants. (n = 24). **(C)** Average relative expression values as determined *via* RNA seq in all studied samples for the Fe homeostasis-related genes illustrated in **Figure 1** (73 genes), the genes annotated with the GO term ‘reactive nitrogen species (RNS) metabolic process’ (GO:2001057; 7 genes) and the genes annotated with the GO term ‘response to ethylene (ET)’ (GO:0009723; 36 genes). ‘C2’ and ‘TIR2’: progeny of ancestor plants that were lifelong biweekly treated with water or 500 µM diproline, respectively. ‘C3’ and ‘TIR3’: progeny of untreated C2 and TIR2 plants, respectively. **(A, B)** Asterisks indicate significant differences upon comparison of TIR2 and TIR3 plants with C2 and C3 plants, respectively, using a two-sided heteroscedastic t-test (p < 0.05). Error bars represent the standard error of the mean. **(C)** Expression levels are quantified as log2fold changes, using same-aged, untreated C2 and C3 plants as controls. Only for the ‘4/3 dpt/i’ data, Mg-inoculated plants at 3 dpi were used as control.

To investigate if diproline-IR is heritable, ancestor plants were treated biweekly until seed set. Seeds were collected and the descendants of lifelong diproline-treated or mock-treated plants (TIR2 or C2, respectively) were grown. Kept untreated, TIR2 and C2 progeny plants were again cultivated to obtain seeds to establish a subsequent generation of progeny plants: TIR3 and C3, respectively. All descendants, while never being exposed to diproline, were subjected to an *Mg* susceptibility assay. The number of galls and females appeared significantly reduced when comparing TIR2 to C2 plants (p = 3.43E-2 and p = 2.02E-2, respectively) (**Figure 4A**). The number of females was also found to be significantly lower in TIR3 plants in comparison with C3 plants (p = 1.65E-2) (**Figure 4B**), revealing a transgenerational induced resistance (TIR) phenotype. At no point, lifelong diproline-treated ancestors or their descendants showed any observable changes in growth or yield (**Supporting Information 5**).

To unravel the pathways underlying the resistance of the descendants of diproline-treated plants, RNA seq was conducted on roots of six-, eighteen- and 30-days-old (6do, 18do and 30do, respectively) TIR2 and C2 plants, as well as on 18do TIR3 and C3 plants. Noteworthy, 18do plants are of the exact same age as the ancestor plants harvested at 4 dpt. All differential expression levels, and DEGs of the TIR RNA seq experiment, as well as GO-enrichment and Mapman analyses can be found in **Supporting Information 6**. **Table 2** shows that TIR2 and TIR3 plants were characterized by a general induction of various defense-related pathways, explaining the reduced susceptibility to *Mg* shown in **Figures 4A**, **4B**. Interestingly, a large part of these pathways was already found to be stimulated upon diproline treatment in the ancestor plants (**Table 1**). In addition, a transgenerational convergence was detected across all RNA seq datasets with respect to expression levels of Fe homeostasis-related genes, genes annotated with the GO term ‘reactive nitrogen species metabolic process’ (GO:2001057), and genes involved in ‘response to ethylene’ (GO:0009723) (**Figure 4C**). No such convergence of expression profiles was detected for other defense-related and/or defense-unrelated pathways (**Supporting Information 7**), implying that there is a biologically relevant co-regulation of the Fe-homeostasis, ET-responses and RNS metabolism in diproline-(T)IR.

**Table 2:** Diproline-transgenerational induced resistance (diproline-TIR) in rice is associated with transcriptional induction of various defense-related compounds and pathways.

6do TIR2 *vs*. C2		
GO term/Mapman bin	Term/bin ID	p-value
Peroxidases	Bin 26.12	<1E-20
Secondary metabolism – phenylpropanoids	Bin 16.2	3.13E-3
Redox reactions *via* the glutathione-ascorbate cycle	Bin 21.2	8.49E-3
Jasmonate metabolism	Bin 17.7	1.95E-2

18do TIR2 *vs*. C2		
GO term/Mapman bin	Term/bin ID	p-value
/	/	/

30do TIR2 *vs*. C2		
GO term/Mapman bin	Term/bin ID	p-value
*PR* genes	Bin 20.1.7	4.86E-6
WRKY transcription factors	Bin 27.3.32	1.74E-2

18do TIR3 *vs*. C3		
GO term/Mapman bin	Term/bin ID	p-value
Response to stress	GO:0006950	7.60E-4
Peroxidase activity	GO:0004601	6.22E-3
Defense response	GO:0006952	4.25E-2
Diterpenoid biosynthesis	KEGG:00904	1.20E-2
Phenylpropanoid biosynthesis	KEGG:00940	1.75E-2

Listed is a selection of plant immunity-associated gene ontology (GO) terms that were significantly enriched among the genes significantly induced in the data sets. MapMan bins that were found to be enriched using a WRS test on all expression levels are also shown. ‘C2’ and ‘TIR2’: progeny of ancestor plants that were lifelong biweekly treated with water or 500 µM diproline, respectively. ‘C3’ and ‘TIR3:’ progeny of untreated C2 and TIR2 plants, respectively. Relative expression values were determined *via* RNA seq in rice roots of six-, eighteen- and 30-days-old (6do, 18do and 30do, respectively) TIR2 plants and 18do TIR3 plants, which were themselves never exposed to diproline. Same-aged and untreated C2 and C3 plants were used as respective controls. PR, PATHOGENESIS-RELATED. Complete results of the GO-enrichment and Mapman analyses can be found in **Supporting Information 6**. /, no significantly enriched GO-term or Mapman bin found.

### DNA methylation levels are modified upon diproline-IR establishment, but this effect is not inherited

Epigenetic changes, such as DNA methylation, potentially underlie the TIR phenomenon. To investigate if genome-wide methylation levels are affected by diproline treatment, 5-mC DNA methylation was first assessed in roots and shoots of diproline-treated ancestor plants at 1 dpt and 4 dpt. At 1 dpt, a significant reduction in shoot methylation levels was detected in the foliarly treated ancestor plants (p = 1.95E-2), whereas root DNA methylation was found to be increased in a near-significant manner at this time point (p = 7.78E-2). Interestingly, a similarly contrasting methylation pattern was also demonstrated upon foliar application of 1 mM β-aminobutyric acid (BABA) at 1 dpt in rice (**Supporting Information 8**), which also corresponds with the time and tissue-dependent fluctuations observed by Kuźnicki et al. (2019) in potato. At 4 dpt, significant hypomethylation was observed in shoots and roots upon diproline treatment (p = 4.59E-3 and p = 2.93E-2, respectively), indicating a specific spatiotemporal dynamic for this epigenetic hallmark upon IR establishment, potentially leading to an expression-permissive open chromatin structure.

**Figure 5:**
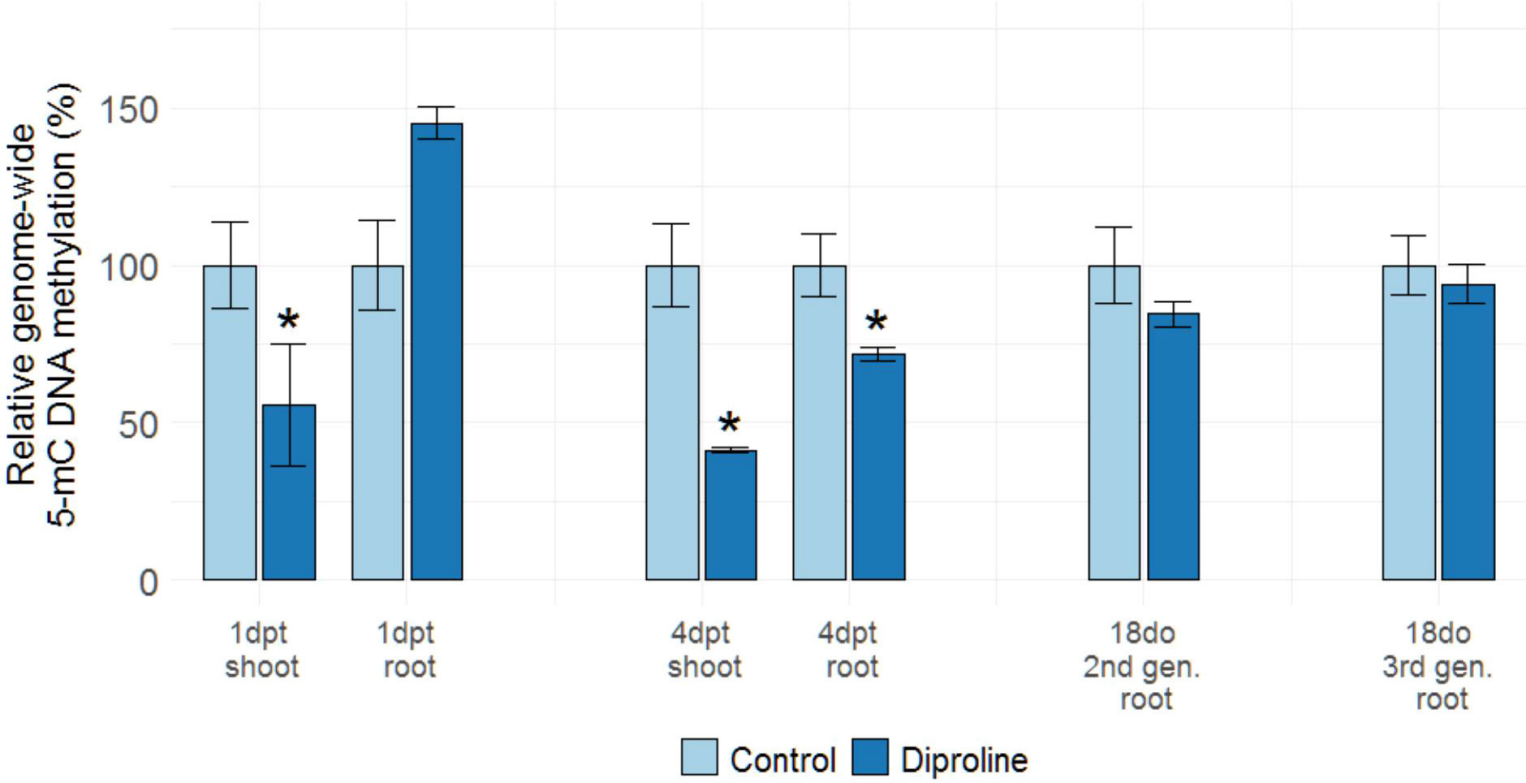
DNA methylation levels in rice roots and shoots upon diproline treatment and in TIR plants. Genome-wide 5-mC DNA methylation levels as assessed by ELISA assays are shown and expressed relative to methylation levels of uninoculated, same-aged and mock-treated or untreated control plants. For the analysis on ancestor plants one and four days post treatment (1 dpt and 4 dpt, respectively), fourteen-days-old rice plants were foliarly treated with 500 µM diproline, while mock-treated plants were used as controls. All second and third generation progeny plants remained untreated and were studied when being eighteen days old (18do). Error bars represent the standard error of the mean. Asterisks indicate significant differences determined *via* a two-sided heteroscedastic t-test (p < 0.05).

To investigate if diproline-TIR is also associated with affected genome-wide DNA methylation levels, root methylation levels were studied in 18do C2, TIR2, C3 and TIR3 samples. We chose specifically for this time point, as the age of the plants in this case corresponds with the 4 dpt time point, where we found consistent DNA hypomethylation in the first generation. No significant differences in 5-mC methylation were detected when comparing roots of 18do TIR2 and TIR3 plants to corresponding control C2 and C3 plants (**Figure 5**). These data indicate that diproline treatment leads to systemic, time-dependent, but non-heritable effects on genome-wide DNA methylation levels.

## Discussion

Foliar treatment with the diketopiperazine diproline had previously been demonstrated to trigger local IR in tobacco, resulting in reduced susceptibility to the leaf pathogens *Phytophthora nicotianae* and tobacco mosaic virus (Wu et al., 2017b). Using our screening platform to identify IR stimuli using rice cell suspension cultures (De Kesel et al., 2020), we had identified diproline (*cyclo*(l-Pro-l-Pro)) as trigger of immunity marker genes and novel IR stimulus that protects rice against the root-knot nematode *Meloidogyne graminicola* (*Mg*) (De Kesel et al., 2020). In the current study, RNA seq data revealed that various defense-related genes are systemically induced by diproline treatment, either leading to direct transcript accumulation or being primed for activation. In case of priming, the defense genes are faster or more strongly activated only when the plants are under attack (De Kesel et al., 2021). Diproline is known to act as attraction pheromone for the diatom *Seminavis robusta* and has been detected in various other species (Bonneure et al., 2021). Possibly, diproline-synthesizing species act as nutritional competitors for rice plants and therefore their presence needs to be detected. Alternatively, the molecular resemblance of diproline to quorum sensing signals of pathogenic microbes (Holden et al., 1999; Von Bodman et al., 2003; Walker et al., 2004; Lee et al., 2010) might explain the immunity-stimulating effect of this molecule. Indeed, microbial quorum sensing signals have already been found to stimulate IR (Khan et al., 2019; Shrestha et al., 2020).

Our results reveal a central role for Fe homeostasis, NO metabolism, redox reactions and hormone signaling in diproline-IR (**Table 1**, **Figure 1**). Fe homeostasis disturbances can modulate plant immunity in various ways. First, it is known that elevated rates of Fe import affect the intracellular redox state by catalyzing the Fenton reaction (Mittler, 2017; Herlihy et al., 2020). The formed ROS can trigger programmed cell death, an effective strategy against biotrophic pathogens (Aznar et al., 2015; Romera et al., 2019), such as *Mg* (Htay et al., 2016). Secondly, *in planta* Fe homeostasis modifications seem to already be sufficient to establish IR (Trapet et al., 2021). Thirdly, increased Fe uptake allows plants to withhold this essential micronutrient from pathogens, impairing them from achieving full virulence (Dellagi et al., 2005; Verbon et al., 2017). A causal relation between disturbed Fe homeostasis and diproline-IR was demonstrated by an *Mg* infection experiment under limited Fe supply (**Figure 2A**) showing that diproline was only effective under sufficient Fe supply. Excessive or very limited Fe provision has detrimental effects on rice development and growth (Aung and Masuda, 2020), which would dramatically impact nematode infection and hence could jeopardize adequate interpretation. Therefore, increased or strongly limiting Fe supplies were not investigated. Although PS secretion was increased upon diproline treatment (**Figure 2B**), this did not affect root Fe levels (**Figure 2C**). This may be explained by the limited capacity of rice to retain Fe in its roots, a consequence of rapid Fe translocation towards the shoots (Aung and Masuda, 2020).

Knowing that Fe supply is essential for diproline-IR (**Figure 2A**), our research underlines the necessity to take the host plant nutritional status into account, when evaluating IR phenotypes, especially in open field trials. Indeed, in spite of promising prior lab experiments, field studies often result in variable and thus unsatisfactory outcomes (Alexandersson et al., 2016; Yassin et al., 2021). Our research illustrates how a good knowledge on the mechanisms underpinning an IR phenotype is needed to optimize IR implementation in agriculture.

In dicots, Fe homeostasis has previously been linked to signaling molecules and/or regulatory processes such as NO, ET, the glutathione-ascorbate redox system and the phenylpropanoid pathway, all known to play a role in plant immunity (Aznar et al., 2015; Verbon et al., 2017; Romera et al., 2019; Herlihy et al., 2020). Transcriptome analyses indicated that nearly all these elements are activated upon diproline treatment in the monocot rice (**Table 1**), while NO and ET were further confirmed to contribute to diproline-IR *via* biochemical assays and infections experiments (**Figure 3**). The well-described synergisms between ROS and RNS on one hand (Turkan, 2018), and between ET and JA on the other (Li et al., 2019), are consistent with our observations. NO has been shown to be involved in innate defense responses of tomato (*Solanum lycopersicum*) against *Meloidogyne incognita* (Zhou et al., 2015), while we reported earlier that ET and JA are key mediators of rice defense against *Mg* (Nahar et al., 2011). The observed induction of a gene encoding nitrate reductase *NIA1* and systemic nitrite accumulation upon diproline treatment in rice confirms and extends the work of Wu et al. (2017b), who detected increased NO levels in guard cells of tobacco leaves upon diproline treatment. A link between Fe-deficiency and ET/NO-induction has also been observed in dicots (Garcia et al., 2011). Similar to Romera et al. (2019) and Herlihy et al. (2020) our data suggest that NO and ET, triggered upon Fe deficiency responses and/or defense stimulation, induce JA and ROS production and detoxification *via* the glutathione-ascorbate redox system.

To study transgenerational IR (TIR), ancestor plants were biweekly treated with diproline to obtain second generation descendants (TIR2) and these plants were used to generate third generation progeny (TIR3). In comparison with descendants of mock-treated plants (C2 and C3), TIR2 and TIR3 plants displayed lower susceptibility to *Mg* (**Figures 4A, B**), while none of these descendants were ever treated with diproline. TIR has only been described once in a monocot plant before: the progeny of benzothiadiazole- or saccharin-treated barley plants was shown to be less susceptible to the leaf pathogen *Rhynchosporium commune* (Walters and Paterson, 2012). Transcriptome analyses revealed a remarkable convergence between the processes that are induced in treated plants and their untreated progeny (**Table 1**, **Table 2**, **Figure 4D**). To our knowledge, such genome-wide transgenerational transcriptional ‘memory’ has not yet been demonstrated for (T)IR phenotypes and might be linked to epigenetic processes. IR and TIR has been associated with genome-wide alterations in DNA methylation levels in dicots (Luna and Ton, 2012; López Sánchez et al., 2016; Stassen et al., 2018; Kuźnicki et al., 2019; López Sánchez et al., 2021). The DNA methylation changes observed upon diproline-treatment in rice fits within the growing body of evidence for IR triggering and possibly inheritance being orchestrated by DNA (de)methylation (Luna and Ton, 2012; López Sánchez et al., 2016; Stassen et al., 2018; Kuźnicki et al., 2019; López Sánchez et al., 2021). The hypomethylated DNA state observed at 4 dpt (**Figure 5**) corresponds with a genome-wide induction of euchromatin, which is associated with transcriptional activation of numerous genes at this time point (**Table 1**, **Supporting Information 4**). Similar to the observations of Kuźnicki et al. (2019) in BABA-treated potato leaves, this global DNA hypomethylation profile is however not inherited by the subsequent generations. If DNA methylation or other epigenetic marks at specific loci is affected in TIR plants warrants further investigation.

In conclusion, our data reveal that diproline-IR against *Mg* in rice is associated with disturbances in the Fe, ET and NO pathways. The IR effect is dependent on sufficient Fe supply and ET-signaling in the first generation. Transgenerational experiments reveal that the IR phenotype and the underlying molecular mechanism is inherited by at least two successive generations. With this study we provide compelling evidence that TIR exists in rice, although the epigenetic or physiological basis for this inheritance remains to be further investigated.

## Experimental procedures

### Plant growth and diproline treatment

*Oryza sativa* ssp. *japonica* cv. Kitaake plants were used for most experiments. The *ETHYLENE- INSENSITIVE PROTEIN 2* (OsEIN2; Os07g0155600)-RNAi line was provided by Yinong Yang (Pennsylvania State University), while a mutant in *CATALASE C* (OsCATC; Os03g0131200) was kindly provided by Lin et al. (2012). In experiments with these lines, *Oryza sativa* ssp. *japonica* cv. Nipponbare was used as wild-type control to match genetic backgrounds. Seeds were germinated for four days at 30°C on wet paper cloths. Seedlings were transferred into PVC tubes containing a mixture of quartz sand (M32; Sibelco; Antwerp, Belgium) and a water absorbing polymer (1003527; De Ceuster Meststoffen (DCM); Grobbendonk, Belgium) to be grown at 28°C in a plant growth room under a 16 h/8 h light/dark regime as described by Sannier et al. (1999). Three times a week, 10 mL of a nutrient solution was given to each plant (**Supporting Information 1**). This solution contained 75 µM iron (Fe), a concentration that was determined *via* in-house optimization for optimal rice growth under our conditions.

In all experiments, plants were treated with diproline at fourteen days after transfer to the substrate. A solution of 500 µM diproline containing 0.02% (v/v) Tween20 (P1379; Sigma-Aldrich; Saint Louis, Missouri, USA) for adequate uptake was foliarly applied *via* a fine mist till run-off. *Via* multiple application rounds with minimal time intervals, 6.25 mL solution was applied per plant. Diproline was synthesized as described earlier (De Kesel et al., 2020). Control plants were mock-treated with Tween20-containing water.

### Susceptibility assays

Nematode inoculation was done on fifteen days-old rice plants, 24 hours after foliar treatment, by applying 250 *Meloidogyne graminicola* (*Mg*) second-stage juveniles (J2s) to each root system. Nematodes were obtained by cutting roots of susceptible rice plants in fine pieces after an infection of approximately three months. Root fragments were laid on a sieve with large mesh (200 µm or larger), which was covered with tissue paper and just immersed in water. This allowed nematodes to migrate through the sieve and accumulate in the collection water. After three days, nematodes were collected on a 25 µm mesh and directly used for inoculation. Plant susceptibility was evaluated 14 days after inoculation by counting galls and nematodes. To visualize galls and nematodes, root systems were boiled for three minutes in a 12.5% raspberry red solution (023.486.4; Alcoferm; Beverlo, Belgium), washed with tap water and destained in acidified glycerol (CL00.0706; Chem-Lab; Zedelgem, Belgium). Galls and nematodes were counted using a stereomicroscope. At least two biologically independent repetitions were conducted per infection experiment, each consisting of eight individual plants per treatment. For the sake of consistency, all datasets were rescaled so that the average #galls/females was the same for each set of eight plants. This normalization led to an average number of fifteen galls, and 40 females per plant. Resulting datasets were then combined and statistical differences were identified using ANOVA and Duncan’s multiple range test or a non-parametric Mann-Whitney test (p < 0.05). As a limited Fe supply can affect plant growth, additional normalization was done for the analysis illustrated in **Figure 2A** by correcting for dried root weight of the plants.

### Nematicidal assay

Nematicidal effects were evaluated by incubating approximately 100 J2s of *Mg* in 1 mL of 500 µM diproline or control solutions. Tap water and Vertimec (Syngenta; Basel, Switzerland) (Pitterna et al., 2009; Li et al., 2018) were used as negative and positive control, respectively. Per time point (*i.e.* two, six and twelve hours of incubation), six biologically independent replicates were studied. Nematode viability was assessed upon contact with a small picking needle. Statistical differences were determined *via* a heteroscedastic two-sided t-test (p < 0.05).

### Effects of lifelong diproline treatment and evaluation of transgenerational IR in rice

Rice plants grown under greenhouse conditions at 28°C (12h light/12h dark) were lifelong biweekly foliarly treated with water or 500 µM diproline as described above, both including Tween20 for adequate uptake. Growth and yield were evaluated in two biologically independent repetitions, consisting of 24 and twelve plants per condition, respectively. The resulting data from the repetitions were combined and normalized so that the control plants were on average 70 cm long, had respectively eight and three tillers and panicles, and resulted in a seed yield of two grams per plant.

Seeds harvested from these first generation biweekly treated plants were used to generate second generation control (C2) and TIR2 plants, respectively. These plants were left untreated and grown under the same conditions as the first generation plants. Seeds harvested on C2 and TIR2 plants were used to grow third generation progeny plants (C3 and TIR3, respectively), again under the same conditions. Noteworthy, these plants were never exposed to diproline themselves. Long-term growth and yield of TIR2 and C2 plants was evaluated in one biologically independent repetition experiment, which included twelve plants per condition. Nematode susceptibility and growth parameters of C2, TIR2, C3 and TIR3 plants were evaluated during infection experiments as described above. For this assay, two biologically independent replicate studies were conducted, each consisting of eight plants per group.

### RNA seq

Transcriptional changes were studied in rice roots *via* RNA seq. For the first generation ancestors, four experimental conditions were analyzed: plants sampled one day post diproline treatment (1 dpt), four days post diproline treatment (4 dpt), three days post inoculation with *Mg* nematodes (3 dpi), and four days post diproline treatment after being inoculated with *Mg* nematodes for three days as well (4/3 dpt/i). As described above, the foliar diproline treatment was done at fourteen days after transfer. Same-aged, mock-treated and uninoculated plants were used as controls. To study transgenerational effects on gene transcription, untreated six-, eighteen- and 30-days-old (6do, 18do and 30do, respectively) C2 and TIR2 plants, as well as 18do C3 and TIR3 plants were assayed. Of note, 18do plants are of the exact same age as the plants of the 4 dpt time point used to study the first generation ancestor plants.

Per condition, three biologically independent replicates were sampled, each consisting of four pooled root systems. RNA extraction was done using the RNeasy Plant Mini Kit (74904; Qiagen; Hilden, Germany) according to the manufacturer’s protocol, with three additional sonication steps of 10 seconds each after addition of the RLT buffer. The QuantSeq 3’ mRNA seq Library Prep Kit (Lexogen; Vienna, Austria) was used for RNA seq library preparation. Quality of the libraries was confirmed using a Bioanalyzer 2100 (Agilent; Santa Clara, California, USA) before being used for sequencing *via* the NextSeq 500 sequencing platform (Illumina; San Diego, California, USA). The samples were multiplexed to minimize lane effects. Single-end reads of 76 nucleotides were generated. Unprocessed sequencing data can be retrieved at NCBI (Geer et al., 2010) as BioProjects PRJNA768961, PRJNA767776 and PRJNA767540 for first, second and third generation plants, respectively. Reads were trimmed with Trimmomatic (version 0.36) using following settings: ILLUMINACLIP : TruSeq3-SE.fa:3:30:10, SLIDINGWINDOW:5:20, MINLEN:20 (Bolger et al., 2014) and mapped against the *Oryza sativa* ssp. *japonica* reference genome (build MSU7.0) using STAR (version 2.5.2a) (Dobin et al., 2013). Only uniquely mapped reads were kept for further analysis. BAM files of multiplexed samples were merged using samtools (version 1.3). Count tables were generated by the ‘Summarize Overlaps’ function in the ‘Genomic Alignments’ R package (version 1.16.0) (Lawrence et al., 2013). Differential expression analysis was performed using the ‘DESeq2’ R package (version 1.20) (Love et al., 2014). A list of differentially expressed genes (DEGs) was made using DESeq2 using an adjusted p-value < 0.05 as cut-off. Gene ontology (GO)-enrichment analyses were done using the g:Profiler tool on biit.cs.ut.ee/gprofiler/gost (Reimand et al., 2016), based on the list of DEGs. Mapman analyses were executed to assess general pathway inductions on all differential gene expression levels (Thimm et al., 2004). Statistical analysis in MapMan was done *via* Wilcoxon Rank Sum (WRS) tests using Benjamini and Hochberg corrected p-values (p < 0.05).

### Detection of diproline in rice roots

Root samples of diproline-treated plants (4 dpt) and same-aged, mock-treated and uninoculated plants (control) were grinded in liquid nitrogen, stored at -20°C and thawed at room temperature before analysis. Per condition and per biological replicate, 100 mg grinded root sample was weighed in a 4 mL glass vial together with 1 nmol caffeine (starting from a stock solution of caffeine in methanol) as internal standard and 2 mL methanol was added. The capped vial, wrapped in aluminum foil, was rotated on a shaker (Edmund Buhler Compact Shaker KS 15A, Bodelshausen, Germany) using low rotation speed for 24 h at room temperature. Subsequently, the extract was filtered with a PES syringe filter (25 mm; 0.2 µm PES membrane; VWR Leuven Belgium; 514-0072) and the filtrate was transferred in an 8 mL glass vial. The 4 mL vial was rinsed with 1 mL methanol, filtered with the PES syringe filter and the filtrate was added to the 8 mL vial. The solvent was removed with a low N_2_ flow, the residue was dissolved in 0.5 mL methanol and transferred to a 1.5 mL vial. The solvent was removed with a low N_2_ flow, the residue was dissolved in 100 µL methanol and transferred to a 150 µL plastic insert in a 1.5 mL vial. The resulting sample was analyzed with gas chromatography-mass spectrometry (GC-MS), using the method of Gillard et al. (2013) (Agilent 8890 GC, Agilent 5977B MSD, splitless mode, 1 µL injection volume, SCAN modus 50-500 (m/z) combined with SIM modus 194 (m/z), GC column: Agilent HP-5ms, 30 m x 250 µm x 0.25 µm).

### Biochemical assays on rice roots

*Phytosiderophores* (PS) - Quantification of Fe binding PS levels in root exudates was done on 4 biological replicates per condition. Each replicate consisted of the pooled root systems of 5 individual plants. Same-aged, mock-treated wild-type plants were used as control. Roots were submerged in 20 mL distilled water for two hours and the exudates were assayed for PS presence *via* the protocol described by Reichman and Parker (2007), who adjusted the protocol of Gries et al. (1995). Small adjustments were made: complexes of Fe^3+^ and PSs were removed *via* Whatman GF/F filters (pore size 0.7 µm; WHA1825021; Sigma-Aldrich; Saint Louis, Missouri, USA) and Fe^2+^ concentrations were determined *via* absorbance measurements at 540 nm using a serial dilution of EDTA (E0511; Duchefa Biochemie; Haarlem, The Netherlands), which can serve as an analogue for PSs in this assay (Reichman and Parker, 2007). Absorbance was measured using an Infinite 200Pro machine (Tecan; Männedorf, Switzerland), using three technical replicates. Finally, PS levels were expressed relative to fresh root weights. For the sake of clarity, PS levels were normalized per time point, leading to a constant PS secretion of 1 nanomole per hour per mg fresh root weight for the controls. Statistical differences were determined *via* a heteroscedastic two-sided t-test (p < 0.05).

*Iron (Fe) levels* - Per condition, three biological replicates were analyzed. Per biological replicate, 100 mg dried tissue was obtained by pooling root material of three plants. Root systems were collected and dried for 72 h at 70°C. Fe levels were quantified as described by (Wu et al., 2017a). Statistical differences were determined *via* a heteroscedastic two-side d t-test (p < 0.05).

*Ethylene (ET) levels* **-** Per condition, five biological replicates were analyzed. Four root systems were pooled per biological replicate. Roots were cut into small pieces and placed in glass vials, which were subsequently sealed to allow ET accumulation in the headspace. After four hours incubation at room temperature, the headspace was analyzed using a Trace 1300 gas chromatograph (Thermo Fisher Scientific; Waltham, Massachusetts, USA) with a flame ionization detector. Statistical differences were determined *via* a heteroscedastic two-sided t-test (p < 0.05).

*Nitric oxide (NO) levels* - Per condition, five biological replicates were analyzed. Six plants were pooled per biological replicate and ground in liquid nitrogen. NO levels were measured using a Nitric Oxide Assay Kit (EMSNO; Thermo Fisher Scientific; Waltham, Massachusetts, USA). This method uses nitrite $\left(NO_{2}^{-}\right)$ levels as a proxy for NO levels, because NO is a highly unstable molecule, while nitrite serves as the precursor of NO in the $NO_{3}^{-}-NO_{2}^{-}-NO$ pathway (Gupta et al., 2011). One hundred mg ground tissue was dissolved in 300 µL of a 100 mM sodium phosphate buffer (pH = 7.4) and cells were lysed by three sonication steps (30 seconds, 30 seconds and 60 seconds, respectively). After centrifuging the samples for 15 minutes at full speed, the supernatant was processed as described in the manufacturer’s protocol. The resulting $NO_{2}^{-}$ levels were determined by taking in account the analyzed ground tissue weight of each sample. Statistical differences were determined *via* a heteroscedastic two-sided t-test (p < 0.05).

*Genome-wide DNA methylation levels* - Eight biologically independent replicates were used per condition, with each replicate consisting of the pooled shoot or root material of three plants. To assess genome-wide DNA methylation (5-mC) levels, the 5-mC DNA ELISA Kit (D5325; Zymo Research; Irvine, California, USA) was used according to the manufacturer’s protocol. Absorbance at 410 nm was measured in a Tecan Infinite 200Pro machine, using two technical replicates. This method was previously shown to provide reliable estimates of genome-wide methylation in rice roots, based on a comparison with whole-genome bisulfite sequencing (Atighi et al., 2020). In this experiment, the effect of foliar treatment with 1 mM β-aminobutyric acid (BABA) was also evaluated in rice at 1 day after treatment, to compare our results with previous observations in potato (Kuźnicki et al., 2019).

## Data availability statement

The datasets presented in this study can be found in online repositories. The names of the repository/repositories and accession number(s) can be found below: https://www.ncbi.nlm.nih.gov/genbank/, PRJNA768961, PRJNA767776 and PRJNA767540.

## Author contributions

Conceptualization: TK, JD. Experimental work: JD, EB, MF. Data analysis: JD, TK, TD and SM. Visualization: JD. Supervision: TK, SM and TD. Writing—original draft preparation: JD. Reviewing: all authors. Funding acquisition, TK. All authors contributed to the article and approved the submitted version.
