# Supplementary material for: Diproline-induced resistance to parasitic nematodes in the same and subsequent rice generations: Roles of iron, nitric oxide and ethylene
Source: Front Plant Sci. 2023 Feb 7;14:1112007. doi: 10.3389/fpls.2023.1112007 (PMC9941634; doi:10.3389/fpls.2023.1112007)
Supplement: Supplementary file 2 [file Table_2.docx]

**A**


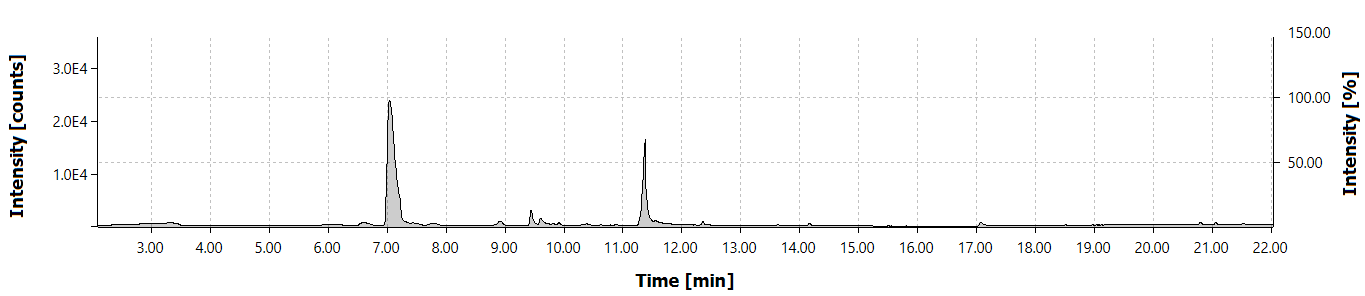


**B**


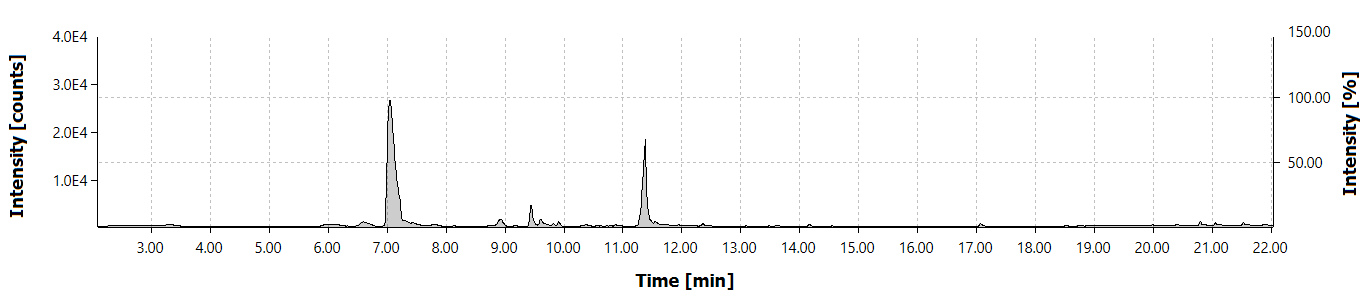


**C**


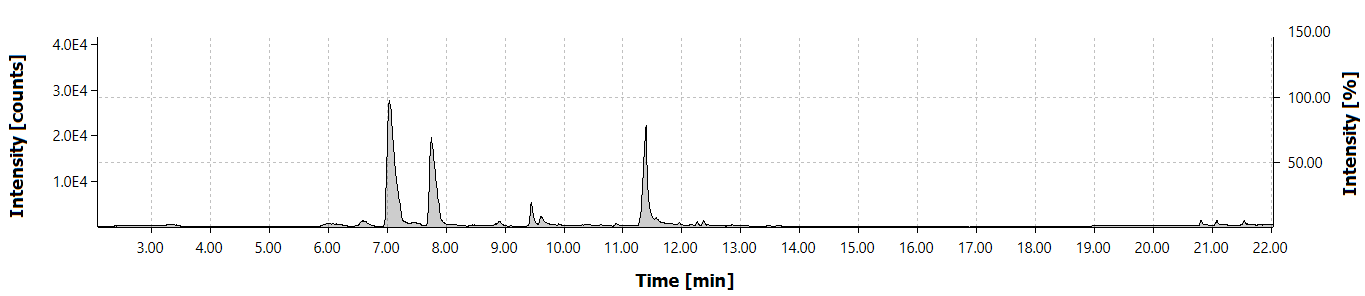


Supporting Information 2: Gas chromatography-mass spectrometry (GC-MS) detection of diproline in rice roots upon foliar treatment. To show that the systemic nature of the diproline-triggered defense induction in rice roots upon foliar treatment was independent of internal diproline transport, detection of diproline in rice roots was attempted at four days past foliar treatment with 500 µM diproline (4 dpt). Same-aged, mock-treated and uninoculated plants were used as control. (a) Representative GC-MS chromatographic profile (SIM modus 194 (m/z), signal of the internal standard caffeine at 7.0 min) of the treated root extract (4 dpt). (b) Representative GC-MS chromatographic profile (SIM modus 194 (m/z), signal of the internal standard caffeine at 7.0 min) of the control root extract (control). (c) Representative GC-MS chromatographic profile (SIM modus 194 (m/z), signal of the internal standard caffeine at 7.0 min) of the extract of control roots (100 mg) spiked with 1 nmol diproline (signal at 7.8 min) before extraction.
