# Supplementary material for: Diproline-induced resistance to parasitic nematodes in the same and subsequent rice generations: Roles of iron, nitric oxide and ethylene
Source: Front Plant Sci. 2023 Feb 7;14:1112007. doi: 10.3389/fpls.2023.1112007 (PMC9941634; doi:10.3389/fpls.2023.1112007)
Supplement: Supplementary file 3 [file Table_3.docx]

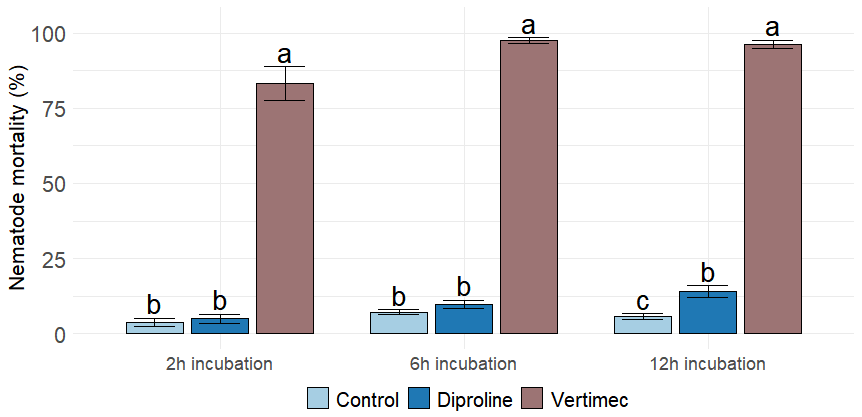


Supporting Information 3: Nematicidal effects of diproline. To assess the nematicidal effects of diproline, 100 second-stage *Meloidogyne graminicola* juveniles were incubated in a solution of tap water (Control), 500 µM diproline or 0.2% (v/v) Vertimec. After two, six and twelve hours of incubation (2hpt, 6hpt and 12hpt, respectively), nematode viability was assessed. Vertimec is a commercial formulation of abamectin and was used as positive control, as it has been demonstrated to be strongly nematicidal (Pitterna et al., 2009; Li et al., 2018). Error bars represent the standard error of the mean. Letters indicate significant differences upon investigation of all possible pairwise comparisons. Statistical differences were determined via a two-sided heteroscedastic t-test (p < 0.05).
