# Supplementary material for: Diproline-induced resistance to parasitic nematodes in the same and subsequent rice generations: Roles of iron, nitric oxide and ethylene
Source: Front Plant Sci. 2023 Feb 7;14:1112007. doi: 10.3389/fpls.2023.1112007 (PMC9941634; doi:10.3389/fpls.2023.1112007)
Supplement: Supplementary file 5 [file Table_5.docx]

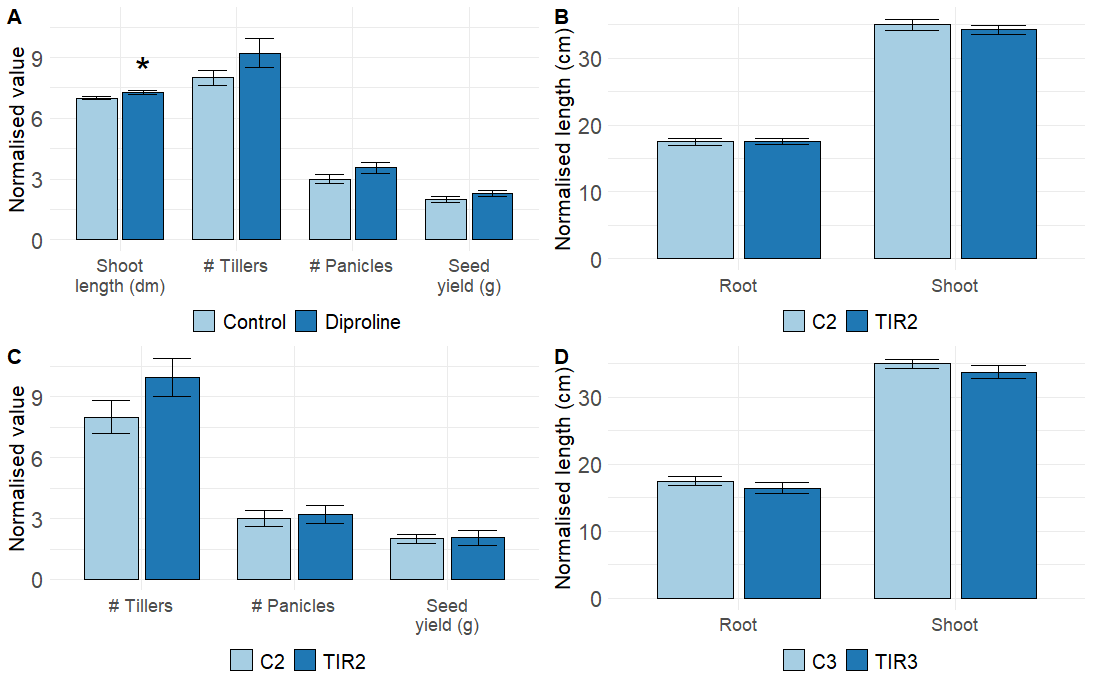


Supporting Information 5: Growth and yield performances of rice plants are not affected by repetitive diproline treatments, nor in plants that have inherited transgenerationally the diproline-induced resistance (diproline-TIR) phenotype. (a) Growth and yield performances of ancestor plants that were biweekly treated with diproline until seed set, as determined when plants were ready for harvest. (b) Growth performances determined in one-month-old C2 and TIR2 plants, determined during infection experiments fourteen days after nematode inoculation. Inoculation took place when plants had reached the age of fifteen days. (c) Growth and yield performances in C2 and TIR2 plants, determined when plants were ready for harvest. (d) Growth performances determined in one-month-old C3 and TIR3 plants, determined during infection experiments fourteen days after nematode inoculation. Inoculation took place when plants had reached the age of fifteen days. Error bars represent the standard error of the mean. Asterisks indicate significant differences when compared to same-aged (a) mock-treated or (b-d) untreated control plants. (a-d) Statistical differences were determined via a two-sided heteroscedastic t-test (p < 0.05). ‘C2’ and ‘TIR2’ refer to the progeny of ancestor plants that were lifelong biweekly treated with water or 500 µM diproline, respectively. ‘C3’ and ‘TIR3’ refer to the progeny of untreated C2 and TIR2 plants, respectively.
