# Supplementary material for: Diproline-induced resistance to parasitic nematodes in the same and subsequent rice generations: Roles of iron, nitric oxide and ethylene
Source: Front Plant Sci. 2023 Feb 7;14:1112007. doi: 10.3389/fpls.2023.1112007 (PMC9941634; doi:10.3389/fpls.2023.1112007)
Supplement: Supplementary file 7 [file Table_7.docx]

Supporting Information 7: Average expression values as determined via RNA seq in all studied RNA seq samples for genes with specific (defense-related) annotations. ‘C2’ and ‘TIR2’ refer to the progeny of ancestor plants that were lifelong biweekly treated with water or 500 µM diproline, respectively. ‘C3’ and ‘TIR3’ refer to the progeny of untreated C2 and TIR2 plants, respectively. Error bars represent the standard error of the mean. Expression levels are quantified as log2fold changes, using same-aged, mock-treated (for 1 dpt and 4 dpt data) or untreated (for TIR2 and TIR3 data) plants. Only for the ‘4/3 dpt/i’ data, *Meloidogyne graminicola*-inoculated plants were used as control. As a result, the ‘4/3 dpt/i’ data illustrate the effects of defense priming (Conrath, 2009; De Kesel et al., 2021). Unlike the parallelism observed in Figure 4d, no resemblance (nor mutual, not with the data illustrated in Figure 4d) can be observed for any of these graphs. Moreover, it should be noted that for the displayed graphs, the average relative expression values are remarkably close to 0, indicating that these processes were, on average, unaffected in the diproline-(T)IR plants. This is in contrast with the results of Figure 4d, which indicate overall up/downregulation of the studied GO terms.
