# Supplementary material for: Diproline-induced resistance to parasitic nematodes in the same and subsequent rice generations: Roles of iron, nitric oxide and ethylene
Source: Front Plant Sci. 2023 Feb 7;14:1112007. doi: 10.3389/fpls.2023.1112007 (PMC9941634; doi:10.3389/fpls.2023.1112007)
Supplement: Supplementary file 8 [file Table_8.docx]

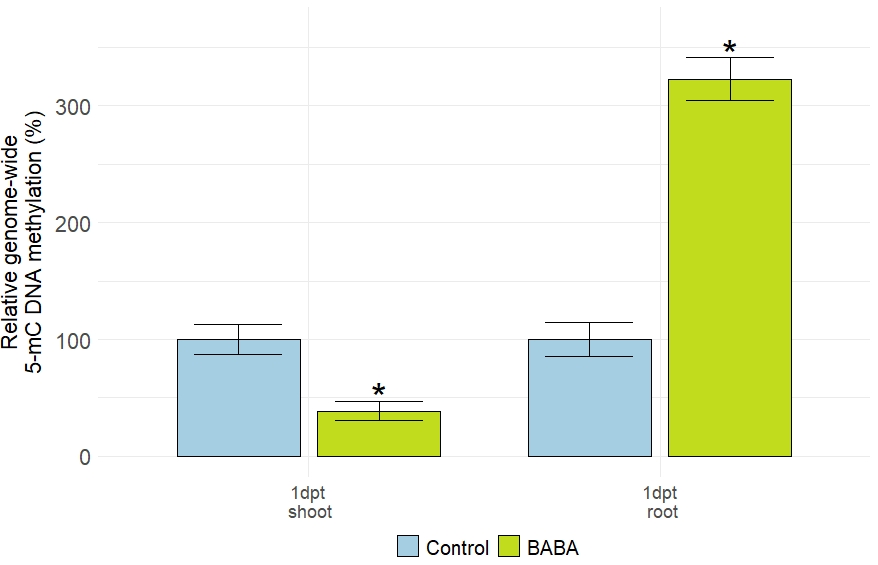


Supporting Information 8: β-amino butyric acid (BABA)-induced resistance (IR) establishment in rice leads to contrasting DNA methylation levels in shoots and roots of rice plants one day post treatment (1 dpt). Genome-wide 5-mC DNA methylation levels as assessed by ELISA assays are shown and expressed relative to methylation levels of uninoculated, same-aged and mock-treated control plants. Fourteen-days-old rice plants were foliarly treated with 500 µM diproline, while mock-treated plants were used as controls. Error bars represent the standard error of the mean. Asterisks indicate significant differences determined via a two-sided heteroscedastic t-test (p < 0.05).
